# Semimetal to semiconductor transition in Bi/TiO_2_ core/shell nanowires†

**DOI:** 10.1039/d0na00658k

**Published:** 2020-12-09

**Authors:** M. Kockert, R. Mitdank, H. Moon, J. Kim, A. Mogilatenko, S. H. Moosavi, M. Kroener, P. Woias, W. Lee, S. F. Fischer

**Affiliations:** Novel Materials Group, Humboldt-Universität zu Berlin 10099 Berlin Germany kockert@physik.hu-berlin.de sfischer@physik.hu-berlin.de; Department of Material Science and Engineering, Yonsei University 03722 Seoul Republic of Korea; Division of Nanotechnology, DGIST 42988 Daegu Republic of Korea; Ferdinand-Braun-Institut, Leibniz-Institut für Höchstfrequenztechnik 12489 Berlin Germany; Laboratory for Design of Microsystems, University of Freiburg – IMTEK 79110 Freiburg Germany

## Abstract

We demonstrate the full thermoelectric and structural characterization of individual bismuth-based (Bi-based) core/shell nanowires. The influence of strain on the temperature dependence of the electrical conductivity, the absolute Seebeck coefficient and the thermal conductivity of bismuth/titanium dioxide (Bi/TiO_2_) nanowires with different diameters is investigated and compared to bismuth (Bi) and bismuth/tellurium (Bi/Te) nanowires and bismuth bulk. Scattering at surfaces, crystal defects and interfaces between the core and the shell reduces the electrical conductivity to less than 5% and the thermal conductivity to less than 25% to 50% of the bulk value at room temperature. On behalf of a compressive strain, Bi/TiO_2_ core/shell nanowires show a decreasing electrical conductivity with decreasing temperature opposed to that of Bi and Bi/Te nanowires. We find that the compressive strain induced by the TiO_2_ shell can lead to a band opening of bismuth increasing the absolute Seebeck coefficient by 10% to 30% compared to bulk at room temperature. In the semiconducting state, the activation energy is determined to |41.3 ± 0.2| meV. We show that if the strain exceeds the elastic limit the semimetallic state is recovered due to the lattice relaxation.

## Abstract 2

Full temperature-dependent thermoelectric and structural characterization of individual bismuth-based core/shell nanowires under the influence of strain induced by the shell.

## Graphical abstract

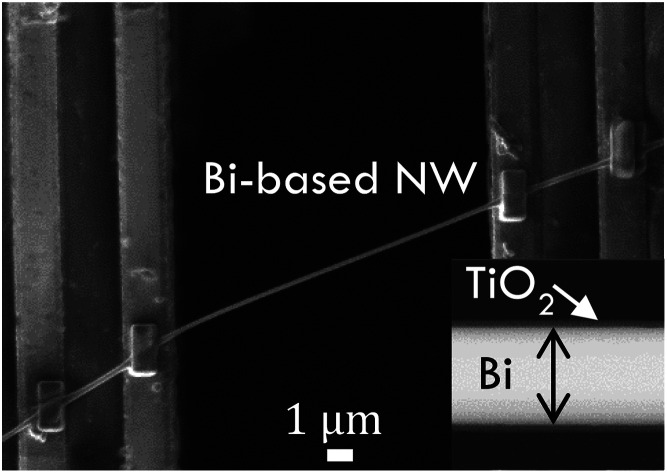

## I. Introduction

Bismuth (Bi) has been under investigations for a long time^1^ due to its unique properties, *e.g.* its anisotropic transport properties, long charge carrier mean free path (up to a few hundred micrometers at 4 K), large Fermi wavelength (70 nm) and semimetal band structure.^1–7^ However, Bi bulk has a low thermoelectric performance,^1,8^ given by the figure of merit 
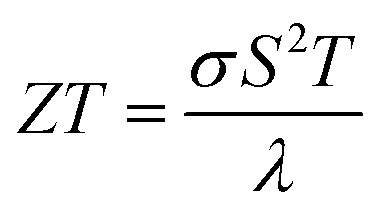
, where *σ* is the electrical conductivity, *S* is the absolute Seebeck coefficient and *λ* is the thermal conductivity at a certain bath temperature *T*. Dresselhaus *et al.*^8^ predicted theoretically an improvement of the thermoelectric efficiency due to quantum size effects for 1D quantum-wire-structures made of Bi. However, the practical implementation of Bi nanowires with such small diameters into applications can be challenging. For this reason, Bi-based core/shell nanowires have raised attention in recent years because of their increased thermoelectric performance for relatively large diameters (*d* > 300 nm).^9–11^ Tellurium (Te) coated as shell on the Bi nanowire core can cause a semimetal to semiconductor transition due to the lattice mismatch of the Bi core and the Te shell.^10,11^ An increased Seebeck coefficient and a reduced thermal conductivity can be the result of such a heterostructure. However, the combined full thermoelectric characterization of individual Bi-based core/shell nanowires, in which all transport parameters (*σ*, *S* and *λ*) are determined on one and the same nanowire, remains an open issue. Here, we demonstrate the full thermoelectric transport and structural characterization of individual core/shell nanowires and investigate titanium dioxide (TiO_2_) as insulating shell material for Bi-based nanowires and compare this to Bi nanowires and Bi/Te core/shell nanowires with a non-uniform shell. We detect the transition from the semiconducting to semimetallic state and *vice versa* and discuss the influence of the shell material on the thermoelectric properties of individual Bi-based nanowires.

## II. Experimental details

The bismuth-based core/shell nanowires consist of a bismuth (Bi) core and a tellurium (Te) or titanium dioxide (TiO_2_) shell, respectively. The single crystalline Bi core was prepared by means of the on-film formation of nanowires (OFFON) method as reported in ref. 9–12. Bi thin films were deposited by radio frequency sputtering on SiO_2_/Si substrates. The sputtering system was evacuated to 10^−7^ Torr before the deposition.^11^ The vacuum was maintained during the sputtering under a 2 mTorr Ar environment at a temperature of 300 K.^11^ After the deposition process, a heat treatment of the Bi thin films was conducted for several hours at 523 K in a vacuum of 10^−7^ Torr.^11^ A compressive thermal stress in the Bi film is induced due to the mismatch of the thermal expansion coefficients of the Bi thin film and the SiO_2_/Si substrate.^12^ This leads to the growth of the Bi nanowires. Radio frequency sputtering was then used to deposit the Te shell onto the Bi core. Atomic layer deposition was used to deposit TiO_2_ as a shell material. All processes were performed in a high vacuum environment to prevent the formation of an oxidation layer between the core and the shell material.

**Fig. 1:**
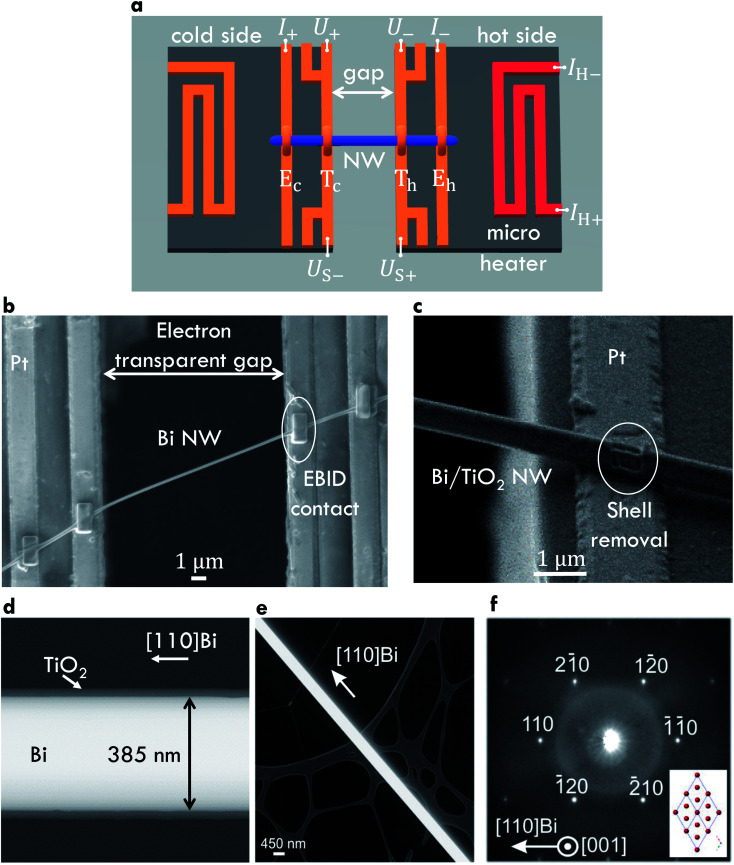
Structural properties of the Bi-based core/shell nanowires. (a) Sketch of the measurement area of the platform. An electron transparent gap divides the measurement area into two sides. The determination of the four-terminal resistance of a nanowire (NW) (blue) can be performed by applying a current *I* at the outer platinum conduction lines *E*_c_ and *E*_h_ (orange) and measuring the voltage *U* at inner conduction lines. The thermovoltage *U*_S_ of a nanowire relative to the platinum conduction lines can be measured by applying a heating current *I*_H_ at the micro heater (red). This creates a temperature difference along the nanowire that can be calculated by four-terminal resistance thermometers *T*_c_ and *T*_h_ (orange) for the cold and hot side, respectively. (b) Scanning electron microscopy image of a Bi nanowire (Bi 1) placed on the thermoelectric nanowire characterization platform. Electrical and mechanical connection between the nanowire and the measurement platform was prepared by electron beam-induced deposition (EBID). (c) Scanning electron microscopy image of a Bi/TiO_2_ nanowire after a selected area shell removal in order to prepare EBID contacts directly at the Bi core. (d) Scanning transmission electron microscopy image of a Bi/TiO_2_ showing the uniform shell thickness. (e) Scanning transmission electron microscopy image of a Bi/TiO_2_ nanowire placed on a carbon film showing the nanowire growth direction. (f) Selected area electron diffraction confirms the single crystalline crystal structure of the Bi core of a Bi/TiO_2_ nanowire.

A thermoelectric nanowire characterization platform (TNCP)^13,14^ was used to perform a full analysis of the transport properties of individual Bi-based core/shell nanowires. An electron transparent gap (width: 10 μm to 20 μm) serves as thermal insulation of the suspended nanowire and enables an investigation of the specimen by scanning (SEM) and transmission electron microscopy (TEM). The measurement zone is situated in the middle of the platform. A sketch of this area is given in Fig. 1a. The TNCP consists of an insulating silicon dioxide surface. On top of that surface, 200 nm platinum electrodes were prepared by radio frequency sputtering. Individual Bi-based nanowires were picked up from the growth substrate and placed on the measurement zone of the TNCP by a micromanipulation system with a thin tungsten tip. Electron beam-induced deposition (EBID) of platinum-based or tungsten-based precursor contacts was conducted in order to prepare a mechanical and electrical connection between the nanowire and the TNCP. For Bi and Bi/Te nanowires, the EBID contacts were applied directly on the shell material, see Fig. 1b. For Bi/TiO_2_ core/shell nanowires, the shell was removed selectively by means of focused ion beam milling before the deposition of the EBID contacts, see Fig. 1c.

A four-terminal configuration of the platinum electrodes is depicted as *E*_c_, *E*_h_, *T*_c_ and *T*_h_ in Fig. 1a. It was used to measure the resistance *R* of the Bi-based nanowires. The electrical conductivity *σ* of Bi-based nanowires can be determined under the assumption that the cross-sectional area of the nanowires is circular by1
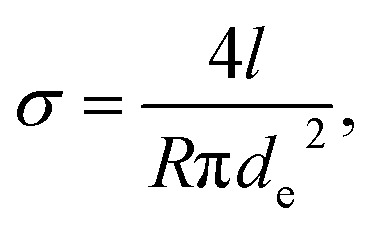
where *R* is the four-terminal resistance of the nanowire, *l* is the length and *d*_e_ is the effective diameter. The effective diameter (*d*_e_ = *d* − 2*t*_shell_, *t*_shell_ is the shell thickness) is smaller than the entire diameter *d* due to the electrical insulating native oxide layer of the Bi nanowires and the electrical insulating TiO_2_ shell of the Bi/TiO_2_ nanowires. The uncertainty of the electrical conductivity *σ* mainly comes from the determination of the geometry parameters. The diameter was measured by scanning electron microscopy (SEM) at several points along each nanowire. The uncertainty of the diameter results from the resolution limitation of the SEM investigations and from the diameter variation of the nanowires and is between 5 nm and 20 nm. The length *l* was also measured by SEM. The uncertainty of *l* mainly comes from the size of the contact area that is defined by the electron beam-induced deposition contacts and varies between 0.4 μm and 1.8 μm. The four-terminal resistance *R* was determined by linear fits of corresponding linear *I*–*V* curves and its relative uncertainty is less than 1%. All geometry parameters are given in Table 1. The resistance of the Bi-based nanowires as a function of the bath temperature is given in the ESI.†

**Table 1:** Geometry parameters. Overview of entire diameter *d*, length *l* and shell thickness *t* of bismuth (Bi) and bismuth/titanium oxide (Bi/TiO_2_) nanowires, respectively. Bi nanowires have a native oxide layer of 5 nm to 10 nm. Bi/TiO_2_ nanowires are coated with a uniform TiO_2_ shell with a thickness of 30 nm. The geometry parameters have been determined by scanning and transmission electron microscopy

Sample	Diameter *d* (nm)	Length *l* (μm)	Shell thickness *t* (nm)
Bi 1	170 ± 5	15.0 ± 0.6	—
Bi 2	210 ± 5	15.4 ± 0.7	—
Bi 3	550 ± 10	11.3 ± 0.7	—
Bi/TiO_2_ 1	155 ± 5	17.1 ± 1.8	30
Bi/TiO_2_ 2	470 ± 10	15.4 ± 1.1	30
Bi/TiO_2_ 3	590 ± 10	15.5 ± 1.3	30

The temperature-dependent thermovoltage *U*_S–Bi-based,Pt_ of individual Bi-based nanowires relative to 200 nm thick platinum conduction lines was measured between bath temperatures of 10 K and 350 K. The temperature difference δ*T* between the hot and the cold side of the nanowires was calculated by the change of four-terminal-resistance thermometers due to the variation of the power of the micro heater on the TNCP by increasing the applied heating current *I*_H_ from zero to −*I*_H,max_ and from zero to +*I*_H,max_ stepwise in equidistant steps. The slope of the function *U*_S–Bi-based,Pt_(δ*T*) gives the relative Seebeck coefficient *S*_Bi-based,Pt_ of the Bi-based nanowires with respect to the platinum conduction lines2
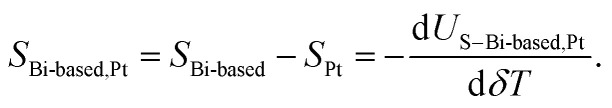


The absolute Seebeck coefficient of a Bi-based nanowire is given by

3 *S* = *S*_Bi-based_ = *S*_Bi-based,Pt_ + *S*_Pt_,

where *S*_Pt_ is the absolute Seebeck coefficient of the platinum reference material. For bath temperatures between *T* = 10 K and *T* = 300 K, *S*_Pt_ was determined in a separate experiment^15^ by measuring a bulk gold wire with known absolute Seebeck coefficient relative to a thin platinum conduction line. For bath temperatures above *T* = 300 K, *S*_Pt_ was taken from bulk platinum.^17,18^ This is reasonable because the difference between *S*_bulk_ and *S*_film_ is within the measurement limit. The uncertainty of the thermovoltage is given by the confidence interval of the thermovoltage which was measured ten times at each step of the applied heating current and then arithmetically averaged. The uncertainty of the relative Seebeck coefficient is determined by the modulus of the largest deviation of the mean value of different fit lines due to applied heating current *I*_H_ that was varied from zero to −*I*_H,max_ and from zero to +*I*_H,max_. The uncertainty of the absolute Seebeck coefficient was determined by error propagation.

The temperature-dependent thermal conductivity *λ* of individual Bi-based nanowires was determined by the change of the resistance of the nanowires due to self-heating.^19^ A current was applied at the outer electrodes *E*_c_ and *E*_h_, see Fig. 1a, and gradually increased. The thermal conductivity *λ* is given by4
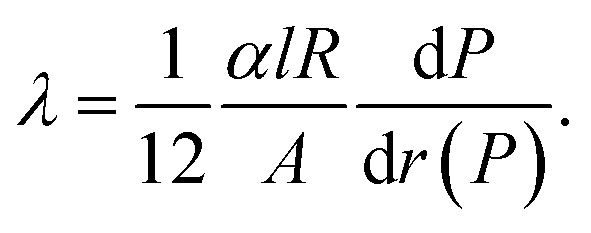
*α* is the temperature coefficient of the resistance of the nanowire, *R* is the four-terminal resistance, *l* is the length, *A* is the cross-sectional area of the nanowire, *P* is the resulting power in the nanowire based on the voltage drop due to the applied current and *r* is the resistance of the nanowire at a certain power. The uncertainty of the thermal conductivity due to the different shell materials depends on the thermal conductivity of the shell material and the cross-sectional area occupied by the shell and will be discussed later.

In the experiments, the four-terminal resistance of the nanowires was measured by a Keithley 6221 AC and DC Current Source and a Keithley 2182A Nanovoltmeter. For Seebeck measurements, the micro heater power was controlled by a Keithley SourceMeter 2401. The thermometer resistances were determined by four-terminal measurements performed by Keithley 6221 and 2182A devices. The thermovoltage was measured by a Keithley 2182A Nanovoltmeter. The measurement configurations were changed by a Keithley 7001 switch matrix system. The transport experiments of the Bi/Te and Bi/TiO_2_ core/shell nanowires were performed in a flow cryostat in helium atmosphere at ambient pressure for the electrical and Seebeck measurements and in vacuum for the thermal conductivity measurements, respectively. All transport experiments of the Bi nanowires were performed in a closed cycle cryocooler in vacuum. Scanning (SEM) and transmission electron microscopy (TEM) as well as energy-dispersive X-ray spectroscopy (EDX) were performed to investigate the structure and chemical composition of the Bi-based core/shell nanowires.

## III. Results and discussion

### A. Structural properties

Fig. 1b shows a Bi nanowire bridging the platform gap and attached with EBID contacts to four platinum conduction lines. Fig. 1c shows a SEM image of a Bi/TiO_2_ core/shell nanowire before the contact preparation. The shell was removed selectively by focused ion beam milling because of the electrical insulating behavior of TiO_2_. Fig. 1d shows an image of a Bi/TiO_2_ nanowire with a uniform shell prepared by atomic layer deposition. A scanning transmission electron microscopy image (Fig. 1e) of a Bi/TiO_2_ nanowire placed on a carbon film exhibits the growth direction. Fig. 1f depicts the selected area electron diffraction pattern of the Bi/TiO_2_ nanowire proving its single crystallinity. Indexing the electron diffraction spots confirms the rhombohedral crystal structure of the Bi core (see the structural model in the inset of Fig. 1f). The geometry parameters of the Bi and Bi/TiO_2_ nanowires are given in Table 1. For comparison, our measurement data for Bi/Te nanowires are given in the ESI.†

**Fig. 2:**
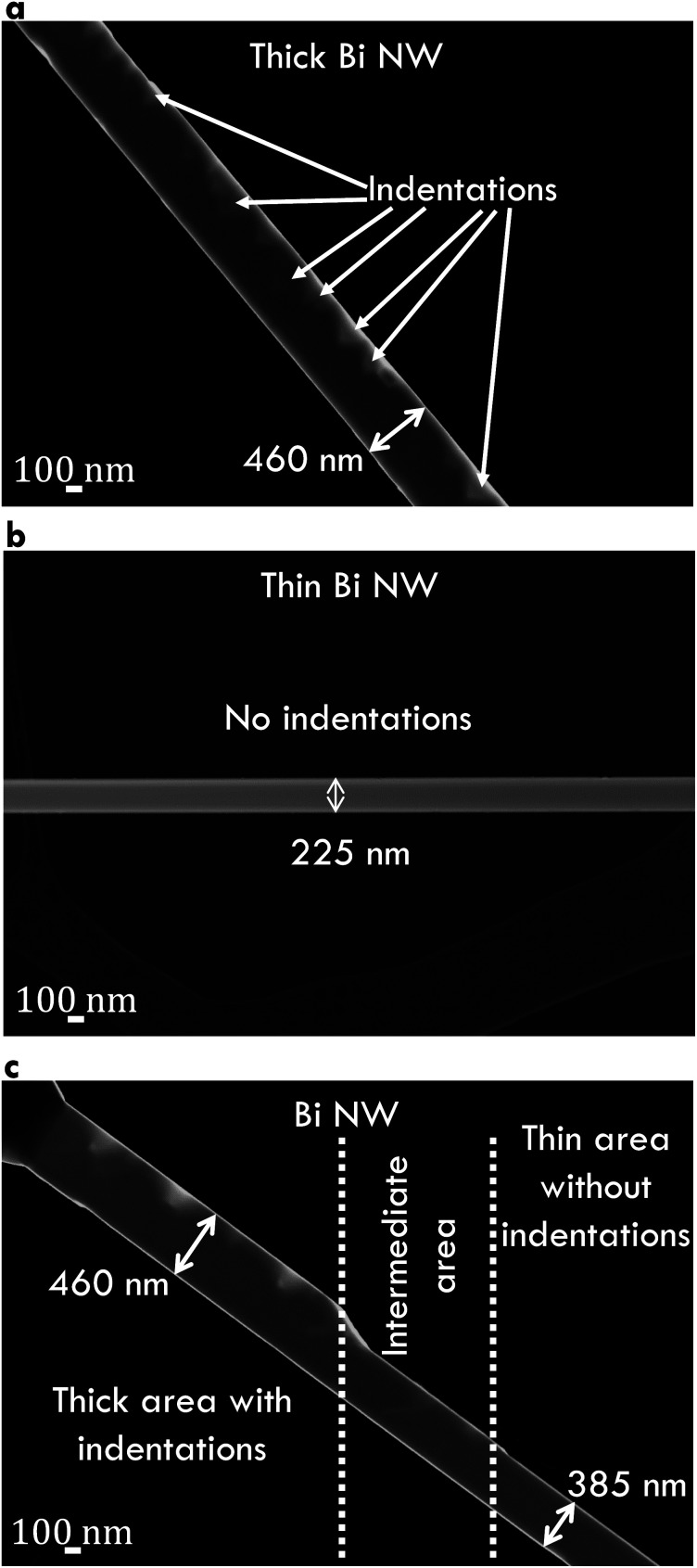
Annular dark-field transmission electron microscopy images of Bi nanowires. (a) Thick nanowire with a rough surface and indentations. (b) Thin nanowire with a smooth surface and no indentations. (c) Bi nanowire with changing diameter. The thick area exhibits several indentations while the thin area is without indentation.

Several Bi nanowires were placed on lacey carbon film in order to investigate the structural properties by transmission electron microscopy. Annular dark-field images of Bi nanowires are given in Fig. 2. Fig. 2a shows a thick (diameter *d* ≈ 460 nm) Bi nanowire with a rough surface and with deep indentations (up to 170 nm deep). In contrast, Fig. 2b shows a thin (*d* ≈ 225 nm) Bi nanowire with a smooth surface without indentations. Both surface properties can be observed on the same Bi nanowire in Fig. 2c. The thick area shows a rough surface with indentations. The diameter of this nanowire changes from thick to thin after several hundred nanometers. Along with this change in diameter, the surface becomes smoother.

The process that leads to the rough surface with indentations of the Bi nanowires may be explained by the different thermal compressive stress between the substrate and the nanowire during the growth process.

### B. Electrical characterization

**Fig. 3:**
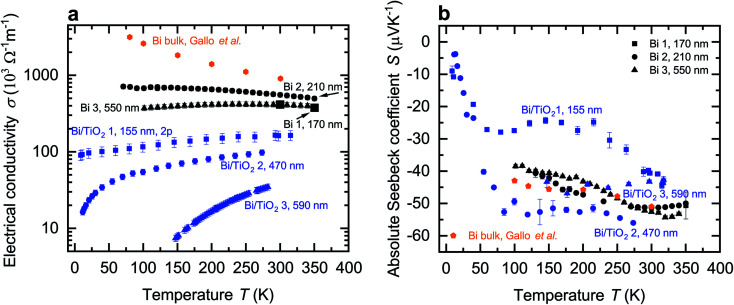
Electrical conductivity and absolute Seebeck coefficient of the Bi-based core/shell nanowires. (a) Electrical conductivity *σ* of the Bi-based core/shell nanowires as a function of the bath temperature *T*. Bi nanowires exhibit a semimetallic-like electrical conductivity whereas the Bi/TiO_2_ nanowires show a semimetallic or seminconducting trend. In addition, the electrical conductivity of Bi bulk (perpendicular to the trigonal axis) from ref. 1 is added. (b) Absolute Seebeck coefficient *S* of the Bi-based core/shell nanowires as a function of the bath temperature *T*. The absolute Seebeck coefficient of Bi bulk (perpendicular to the trigonal axis) from ref. 1 is added.

The temperature-dependent electrical conductivity *σ* of Bi and Bi/TiO_2_ nanowires is shown Fig. 3a. Moreover, *σ*_bulk_ (perpendicular to the trigonal axis) from ref. 1 is added to the diagrams. The electrical conductivity of all nanowires is reduced compared to the bulk material. Bi/TiO_2_ nanowires exhibit a clearly reduced electrical conductivity compared to the Bi nanowires and a semiconducting temperature dependence of the electrical conductivity.

The temperature dependence of the electrical conductivity of the bulk Bi semimetal can be explained by the competing influence of carrier concentration and mobility.^1,5,6,20–22^ Bi bulk has a small carrier concentration varying between 2.7 × 10^17^ cm^−3^ to 3.0 × 10^18^ cm^−3^ at temperatures between 2–300 K.^1,5,6,22^ The change of mobility *μ* of Bi bulk exceeds the change of the carrier concentration by more than one order of magnitude in the temperature range from 77–300 K.^1,5,6^

All Bi nanowires show semimetallic behavior and have a reduced electrical conductivity compared to that of bulk. This can be attributed to enhanced surface scattering due to a higher surface-area-to-volume ratio in nanowires. Hence, *σ* of Bi 1 (170 nm) is reduced compared to that of Bi 2 (210 nm). A smaller diameter leads to increased surface scattering, which will reduce the electrical conductivity, because the mean free path of the charge carriers becomes restricted by the nanowire diameter. This is consistent with ref. 21, 22 and 25.

However, *σ* of Bi 3 (550 nm) is reduced compared to that of Bi 2 (210 nm) despite the larger diameter. The temperature dependence of the electrical conductivity of Bi 3 (550 nm) can be attributed to a change of the dominant scattering mechanism from surface scattering (Bi 1 (170 nm) and Bi 2 (210 nm)) to scattering at indentations (Bi 3 (550 nm)). Additional scattering at indentations arises preferably in nanowires with larger diameters as shown in Fig. 2.

According to Matthiessen's rule, the total scattering rate, which is given by *τ*_tot_^−1^ = *τ*_bulk_^−1^ + *τ*_sc_^−1^ + *τ*_ind_^−1^, leads to a reduction of the charge carrier mean free path. *τ*_bulk_^−1^ characterizes the inverse lifetime in the bulk material and its temperature dependence can be described by the Bloch–Grüneisen relation. *τ*_sc_^−1^ is the surface scattering rate which depends on the nanowire diameter. *τ*_ind_^−1^ is the indentation scattering rate which depends on the density and depth of the indentations.

For Bi/TiO_2_ core/shell nanowires, an electrical conduction in the shell material can be neglected due to the electrical insulating TiO_2_. All Bi/TiO_2_ nanowires show a decreasing electrical conductivity with decreasing bath temperature. Bi/TiO_2_ core/shell nanowires show an increasing electrical conductivity with decreasing diameter and exhibit a diameter-dependent transition from the semiconducting to the semimetallic state.

**Fig. 4:**
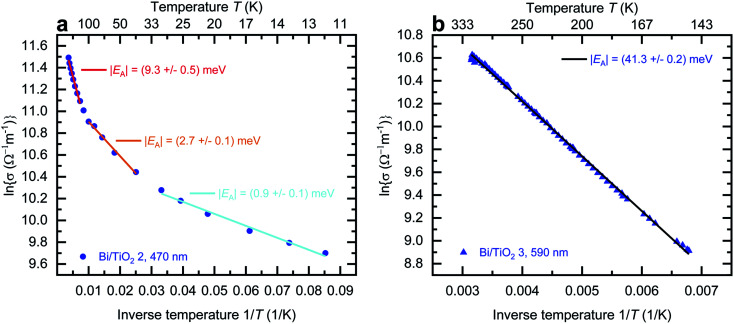
Activation energy of Bi/TiO_2_ core/shell nanowires. (a) Natural logarithm of the electrical conductivity of Bi/TiO_2_ 2 (470 nm) as a function of the inverse bath temperature *T*^−1^. The modulus of the activation energy |*E*_A_| can be determined in three distinct temperature ranges from 10 K to 30 K, from 40 K to 120 K and from 140 K to 270 K. Increasing the bath temperature also increases the activation energy. (b) Natural logarithm of the electrical conductivity of Bi/TiO_2_ 3 as a function of the inverse bath temperature *T*^−1^. The activation energy |*E*_A_| can only be determined in the temperature range from 140 K to 310 K.

The effect of strain on the electrical conductivity can be observed for the Bi/TiO_2_ nanowires. However, there are some different manifestations of the strain influence and the resulting change of the electrical conductivity. The effect of the so-called elastic strain of the TiO_2_ shell on the Bi core can be observed for Bi/TiO_2_ 3 (590 nm) in Fig. 3a. The electrical conductivity of Bi/TiO_2_ 3 (590 nm) is 25 times smaller than that of the bulk material and nearly 15 times smaller compared to the Bi nanowires at room temperature. The strain effect leads to an opening of a band gap. This is illustrated in Fig. 4 that shows the natural logarithm of the electrical conductivity of the Bi/TiO_2_ nanowires as a function of the inverse bath temperature. The Arrhenius equation5
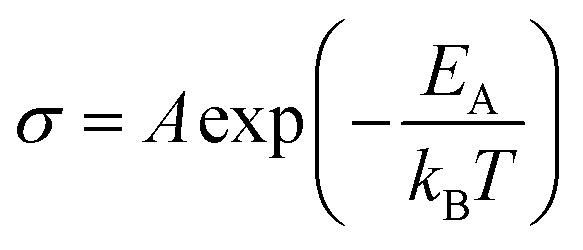
can be used to determine the activation energy *E*_A_. *σ* is the electrical conductivity, *A* is a constant, *k*_B_ is the Boltzmann constant and *T* is the bath temperature. An activation energy of |41.3 ± 0.2| meV in the temperature range from 140 K to 310 K was determined for Bi/TiO_2_ 3 (590 nm). For Bi/TiO_2_ 2 (470 nm), a reduced activation energy of |9.3 ± 0.5| meV compared to Bi/TiO_2_ 3 (590 nm) was determined.

This reduction is accompanied by an increase of the electrical conductivity. This may be attributed to a partly lattice relaxation which leads to the beginning of the recovery of the semimetallic state induced by the semiconducting to semimetallic transition. For this reason, the electrical conductivity of Bi/TiO_2_ 2 (470 nm) is larger than that of Bi/TiO_2_ 3 (590 nm) despite the smaller core diameter.

The lattice relaxation process can be understood in a way that the influence of the shell on the core exceeds the elastic limit and as a result, the Bi core relaxes spontaneously. The probability that this relaxation process occurs is higher for nanowires with smaller diameter because the compressive strain has the largest influence at the interface between the core and the shell and decreases with some distance from the interface as shown in ref. 10 and 11 for Bi/Te core/shell nanowires. For this reason, the largest electrical conductivity can be observed for Bi/TiO_2_ 1 (155 nm) due to the fully relaxed, non-elastic influence of the strain effect. The electrical conductivity of this nanowire could only be determined from two-terminal (2p) resistance measurements. Hence, the values in Fig. 3a denote only a lower limit of the electrical conductivity. This is determined by the contact resistance. However, the tendency of the recovery of the semimetallic state is clearly visible.

**Fig. 5:**
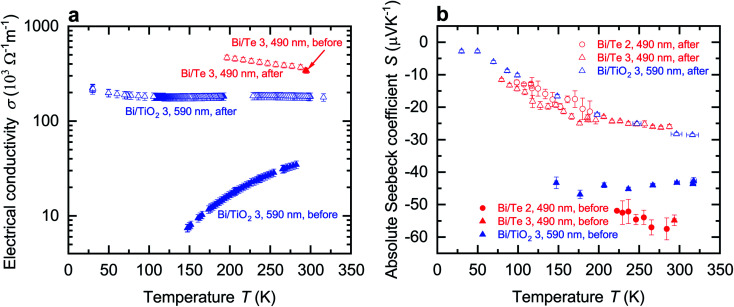
Influence of lattice relaxation on electrical conductivity and absolute Seebeck coefficient of Bi-based core/shell nanowires. *Before*, indicates the corresponding transport properties before the lattice relaxation. *After*, indicates the corresponding transport properties after the lattice relaxation. (a) Electrical conductivity *σ* of the Bi-based core/shell nanowires as a function of the bath temperature *T*. The relaxation process of the core/shell structure of Bi/TiO_2_ 2 (470 nm) leads to a change of the temperature dependence of the electrical conductivity from semiconducting to semimetallic. A small change of *σ* can also be observed for Bi/Te 3 (490 nm). (b) Absolute Seebeck coefficient *S* of the Bi-based core/shell nanowires as a function of the bath temperature *T*. The relaxation process induces a significant change of the absolute Seebeck coefficient of the Bi-based core/shell nanowires. The changes in the transport properties after the relaxation process indicate that the shell had a significant compressive strain effect on the Bi core.

In addition, the lattice relaxation process was observed for Bi/TiO_2_ 3 (590 nm). An irreversible increase of the electrical conductivity occurred during the measurement and the temperature dependence of *σ* changed from semiconducting to semimetallic, see Fig. 5a. This can be understand as evidence of the proposed recovery of the semimetallic state due to lattice relaxation. The relaxation process of the Bi core, which changes the electrical conductivity of Bi/TiO_2_ 3 (590 nm) also leads to a significant and irreversible reduction of the Seebeck coefficient, see Fig. 5b. This was also observed for the Bi/Te nanowires. Additional information of the lattice relaxation process, which is attributed to the different thermal expansion coefficients of the core and the shell, is provided in the ESI.†

For a comparison, the electrical properties of the Bi/Te core/shell nanowires are given in the ESI.†

### C. Thermoelectric characterization

The temperature-dependent absolute Seebeck coefficient *S* of all Bi and Bi/TiO_2_ nanowires is shown in Fig. 3b. *S*_bulk_ (perpendicular to the trigonal axis) from ref. 1 is added to the diagrams. The absolute Seebeck coefficients of all Bi nanowires are comparable with the bulk material in terms of both magnitude and temperature dependence. However, *S* of Bi/TiO_2_ 1 (155 nm) is reduced by 27% compared to the bulk material at *T* = 300 K.

As the absolute Seebeck coefficient *S* of Bi bulk and of all Bi-based nanowires is negative, electrons are identified as the dominant charge carriers. In general, the total Seebeck coefficient *S*_tot_ is determined by the partial contribution of holes and electrons and it is given by6
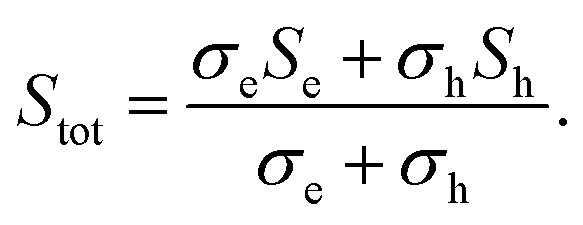
Here, *σ*_e_ and *σ*_h_ are the partial electrical conductivities of the electrons and holes, respectively and *S*_e_ and *S*_h_ are the partial Seebeck coefficients of the electrons and holes, respectively. Theoretical studies revealed that each partial Seebeck coefficient can be larger than *S*_tot_^8,22^ but due to the opposite sign of both contributions, they cancel each other out. This results in a saturation regime with a weak temperature dependence between bath temperatures of *T* = 100 K and *T* = 300 K of *S*_bulk_ and in general also for the Bi-based nanowires. With decreasing bath temperatures, the absolute Seebeck coefficient is expected to tend linear to zero below *T* = 100 K due to the decrease of the thermodiffusion contribution. This temperature dependence of *S* is expected for all investigated Bi-based nanowires as observed for Bi/TiO_2_ 2 (470 nm).

The temperature dependence and absolute value of *S* of the Bi nanowires are comparable with that of bulk.^1^ Theoretical studies showed that Bi nanowires exhibit only a small change of *S* along the binary axis for diameters between *d* = 100 nm and *d* = 500 nm.^26^ A significant change of *S* of the Bi nanowires is expected only for diameters below 60 nm due to a change of the density of states.^22^

For Bi/TiO_2_ core/shell nanowires, a contribution of the shell to the total absolute Seebeck can be neglected due to the electrical insulating TiO_2_. Bi/TiO_2_ 1 (155 nm) has the smallest Seebeck coefficient over a wide temperature regime of all investigated Bi-based nanowires. Furthermore, a larger change of *S* with decreasing bath temperatures compared to Bi bulk and the other Bi-based nanowires can be observed between *T* = 100 K and *T* = 300 K. This can be attributed to the small diameter of the Bi core that is only 95 nm without the shell. The spatial limitation leads to more surface scattering, a reduced charge carrier mean free path and as a result to a reduction of the thermodiffusion contribution (from both electrons and holes) to the absolute Seebeck coefficient.^15,16^ Furthermore, the slightly increase of *S* at around *T* = 100 K may be attributed to the influence of phonon drag.

For Bi/TiO_2_ nanowires with a larger diameter, like Bi/TiO_2_ 2 (470 nm) and Bi/TiO_2_ 3 (590 nm), the absolute Seebeck coefficient is comparable with that of the Bi nanowires. The slight increase of the Seebeck coefficient of Bi/TiO_2_ 2 (470 nm) compared to that of the Bi nanowires and Bi bulk may be attributed to the influence of the compressive strain effect of the TiO_2_ shell on the Bi core which results in the transition of the semimetallic to semiconducting behavior of the electrical conductivity. The accompanied band opening may reduce the partial hole contribution of the bipolar transport to the total Seebeck coefficient, see eqn (6). The further band opening of Bi/TiO_2_ 3 (590 nm) do not lead to an increase of the Seebeck coefficient. However, the electrical conductivity of this nanowire is significantly reduced compared to the electrical conductivity of the other Bi/TiO_2_ nanowires. For this reason, the further pronounced reduction of the weighting factor *σ*_e_ and *σ*_h_, see eqn (6), limit the actual amount of the Seebeck coefficient.

The relaxation process of the Bi core, which changes the electrical conductivity of Bi/TiO_2_ 3 (590 nm), also leads to a significant and irreversible reduction of the Seebeck coefficient, see Fig. 5b. As the degree of band energy overlap increases a transition from semiconducting to semimetallic behavior is induced. This is also observed for the Bi/Te nanowires.

### D. Thermal characterization

**Fig. 6:**
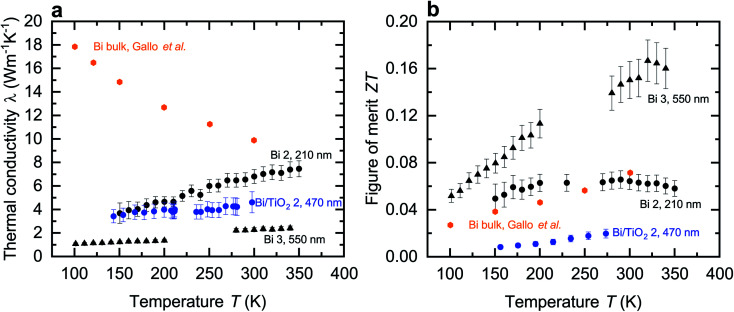
Thermal conductivity and figure of merit of the Bi-based core/shell nanowires. (a) Thermal conductivity *λ* of the Bi-based core/shell nanowires as a function of the bath temperature *T*. The thermal conductivity of Bi bulk (perpendicular to the trigonal axis) from ref. 1 is added. *λ* of the Bi-based core/shell nanowires is reduced compared to the bulk material and shows a monotonic decrease of the thermal conductivity with decreasing bath temperature. (b) Figure of merit *ZT* of the Bi-based core/shell nanowires as a function of the bath temperature *T*. The figure of merit of Bi bulk (perpendicular to the trigonal axis) from ref. 1 is added.

In contrast to the electrical conductivity, all shell materials contribute to the thermal conductivity *λ*. Thus, the entire diameter *d*, as given in Table 1, of the nanowires is required to determine *λ*. Fig. 6a shows the thermal conductivity of the Bi and Bi/TiO_2_ nanowires and *λ*_bulk_ (perpendicular to the trigonal axis) from ref. 1. The thermal conductivity of all nanowires is reduced compared to the bulk and exhibits a monotonic decrease in *λ* with decreasing bath temperature while Bi bulk shows a monotonic increase. In general, *λ* depends on partial contributions from different heat carrier sources and the electronic contribution can be estimated by the Wiedemann–Franz relation.^27,28^ For Bi bulk it has been shown, that phonons are the dominant heat carrier source at low temperatures.^1,29^ As the bath temperatures rises, the charge carrier contribution becomes the dominant part.^1,29^ At *T* = 300 K, nearly 70% of the total thermal conductivity can be attributed to the charge carrier contribution. For Bi-based nanowires, the increased surface-area-to-volume ratio acts both on the charge carrier and lattice scattering which leads to a reduction of the thermal conductivity.

For core/shell nanowires, the shell material has to be taken into account in order to determine the total thermal conductivity *λ*_tot_ as given by7
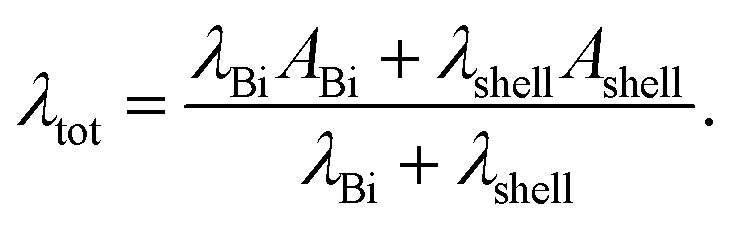
*λ*_Bi_ and *λ*_shell_ are the partial thermal conductivities of the Bi core and of the shell material, respectively. *A*_Bi_ and *A*_shell_ are the partial cross-sectional areas of the Bi core and of the shell material, respectively. An upper limit of the thermal conductivity for bismuth oxide is 2.2 W m^−1^ K^−1^.^23^*λ* of titanium dioxide films is 1.3 W m^−1^ K^−1^.^24^ The thermal conductivity of the shell yields to a relative uncertainty of the thermal conductivity of the complete nanowires ranging from <1% for Bi 3 (550 nm) due to large cross-sectional area of the Bi core compared to the small cross-sectional area of the bismuth oxide shell to 14% for Bi/TiO_2_ 1 (155 nm) due to the larger cross-sectional area of the TiO_2_ shell. The dominant contribution to the thermal conductivity comes from the Bi core. This applies for all core/shell nanowires investigated in this work.

The thermal conductivity of Bi 2 (210 nm) is comparable with other Bi nanowires reported in ref. 22 and 25. The reduction of *λ* and the change of the temperature dependence can be attributed to the spatial confinement of the nanowires and the resulting increased phonon surface and charge carrier surface scattering.^22,25^ The dominant contribution to the thermal conductivity comes from charge carriers, even when the bath temperatures decreases.

Bi 3 (550 nm) has the smallest *λ* of all Bi-based nanowires investigated in this work. Scattering at the rough surface and at indentations, see Fig. 2a, lead to a strong reduction of the thermal conductivity. This reduction can mainly be attributed to a reduction of the lattice thermal conductivity. For this reason, *λ* of Bi 3 (550 nm) is smaller than that of Bi/TiO_2_ 2 (470 nm) despite the larger electrical conductivity of Bi 3 (550 nm).

However, the thermal conductivity of Bi/TiO_2_ 2 (470 nm) is reduced compared to the bulk material and to that of Bi 2 (210 nm). The reduction of *λ* of Bi/TiO_2_ 2 (470 nm) can be attributed to an increase of charge carrier and phonon interface scattering comparable to the effect of the Te shell on the thermal conductivity of Bi/Te core/shell nanowires.^9–11^ The compressive strain effect of the shell, which leads to a reduction of the electrical conductivity, will also lead to a reduction of the charge carrier contribution to the total thermal conductivity.

### E. Figure of merit

The thermoelectric figure of merit *ZT* at a given bath temperature *T* is determined by8
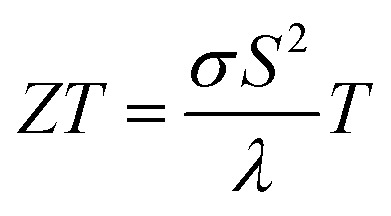
and depicted for the Bi-based nanowires in Fig. 6b. For comparison, the figure of merit of bulk Bi is *ZT* = 0.07 at room temperature.^1^ This is comparable with *ZT* of Bi 2 (210 nm).

Bi 3 (550 nm) has the highest figure of merit *ZT* = 0.15 at room temperature. This can be attributed to the strong reduction of the thermal conductivity that is a result of scattering effects at the rough surface and in addition at indentations. The thermoelectric properties are tunable over a wide range by the choice of the shell material and the nanowire diameter. For a high figure of merit, it is necessary that the Seebeck coefficient is large enough. The Seebeck coefficient can be increased by shifting the Fermi energy and opening a band gap. Bi/TiO_2_ nanowires show a rather low figure of merit. This can be attributed to the significantly reduced electrical conductivity compared to other Bi-based nanowires due to the influence of the TiO_2_ shell on the Bi core.

### F. Conclusion

The full temperature-dependent thermoelectric characterization of individual Bi-based core/shell nanowires shows the influence of the shell material on the electrical conductivity, the absolute Seebeck coefficient and the thermal conductivity. Bi-based nanowires are semimetallic or semiconducting depending on the extent of the compressive strain effect induced by the shell. Scattering of charge carriers at surfaces, indentations and core/shell interfaces leads to a reduction of the electrical as well as the thermal conductivity compared to the bulk material. The compressive strain on the Bi core by the shell can increase the Seebeck coefficient by band opening. However, if the strain exceeds the elastic limits, a relaxation process leads irreversibly to a transition from a semiconducting to semimetallic behavior. As a consequence, Bi-based nanowires can be tailored by a shell in a way that the transport properties are tunable over a wide range.

## Conflicts of interest

There are no conflicts to declare.

## Supplementary Material

NA-003-D0NA00658K-s001
