# Supplementary material for: Semimetal to semiconductor transition in Bi/TiO2 core/shell nanowires
Source: Nanoscale Adv. 2020 Dec 9;3(1):263–71. doi: 10.1039/d0na00658k (PMC9419100; doi:10.1039/d0na00658k)
Supplement: NA-003-D0NA00658K-s001 [file NA-003-D0NA00658K-s001.pdf]

Electronic Supplementary Material (ESI) for Nanoscale Advances.
This journal is © The Royal Society of Chemistry 2020

Supplementary Information - Semimetal to semiconductor transition in Bi/TiO_2 core/shell nanowires

M. Kockert,[1,*] R. Mitdank,[1] H. Moon,[2] J. Kim,[3] A. Mogilatenko,[4] S. H. Moosavi,[5] M. Kroener,[5] P. Woias,[5] W. Lee,[2] and S. F. Fischer[1,†]

[1]*Novel Materials Group, Humboldt-Universität zu Berlin, 10099 Berlin, Germany*
[2]*Department of Material Science and Engineering, Yonsei University, 03722 Seoul, Republic of Korea*
[3]*Division of Nanotechnology, DGIST, 42988 Daegu, Republic of Korea*
[4]*Ferdinand-Braun-Institut, Leibniz-Institut für Höchstfrequenztechnik, 12489 Berlin, Germany*
[5]*Laboratory for Design of Microsystems, University of Freiburg - IMTEK, 79110 Freiburg, Germany*

[*] kockert@physik.hu-berlin.de
[†] saskia.fischer@physik.hu-berlin.de

I. S1: RESISTANCE OF BI-BASED NANOWIRES

Fig. 1a shows the four-terminal resistance of the Bi-based nanowires as a function of the bath temperature T. The Bi nanowires (Bi 1 (170 nm), Bi 2 (210 nm) and Bi 3 (550 nm)) exhibit a semimetallic-like temperature dependence of the resistance. A linear representation of the pronounced temperature dependence of R of Bi 3 is given in Fig. 1b. The Bi/TiO_2 core/shell nanowires show an increase of the resistance with decreasing bath temperatures.

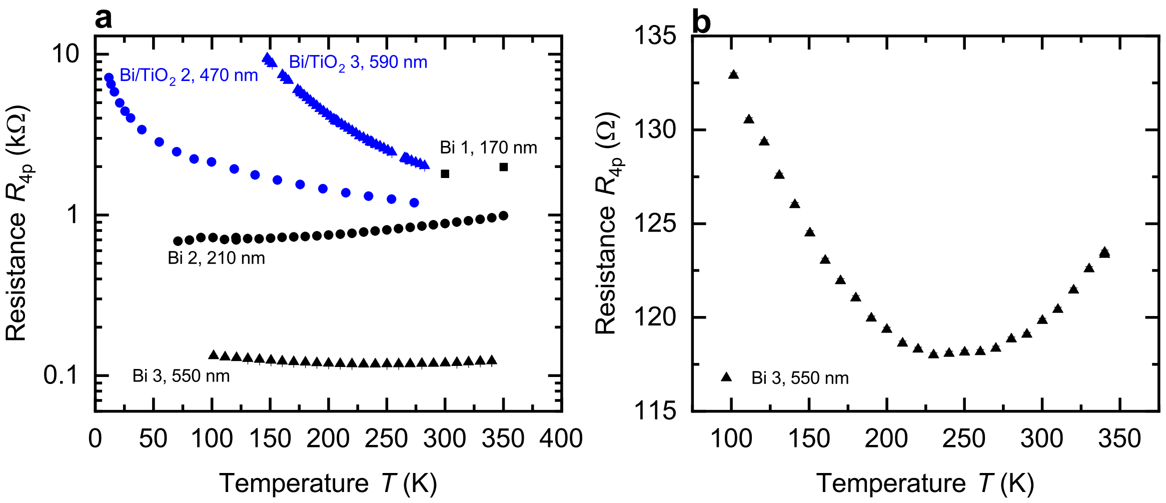

FIG. 1. **Resistance of the Bi-based core/shell nanowires.** **a**, Four-terminal resistance R_{4p} of the Bi-based core/shell nanowires as a function of the bath temperature T. Bi nanowires exhibit a semimetallic temperature dependence. Bi/TiO_2 nanowires show a semiconducting behavior of the resistance. **b**, Four-terminal resistance R_{4p} of Bi 3 (550 nm) as a function of the bath temperature T in a linear representation of $R(T)$. The resistance is decreasing with decreasing bath temperatures from T = 340 K down to T = 270 K. Below T = 230 K, the resistance is increasing with decreasing temperatures. From the temperature dependence of the electrical conductivity the modulus of the thermal activation energy of Bi 3 (550 nm) is determined to $|2.0 \pm 0.1|$ meV in the temperature range from 100 K to 200 K.

II. S2: DETERMINATION OF THE THERMAL CONDUCTIVITY OF BI-BASED NANOWIRES

The thermal conductivity λ of the Bi-based nanowires was determined by the increase of the resistance of the wire due to self-heating [6]. A current was applied at the outer contacts of the nanowire and gradually increased. λ is given by

$$\lambda = \frac{1}{12}\frac{\alpha l R}{A}\frac{\mathrm{d}P}{\mathrm{d}r(P)}. \tag{1}$$

α is the temperature coefficient of the resistance of the nanowire, R is the four-terminal resistance, l is the length, A is the cross-sectional area of the nanowire, P is the resulting power in the nanowire based on the voltage drop due to the applied current and r is the resistance of the nanowire at a certain power. The main contributions to the uncertainty of the thermal conductivity are coming from the determination of the nanowire length, the resistance increase and the temperature coefficient of the resistance.

Fig. 2 shows the resistance r of Bi 2 (210 nm) as a function of the power P in the nanowire due to the applied current at 300 K. The slope of the corresponding curves yields the relation between r and P that is necessary in order to determine the thermal conductivity.

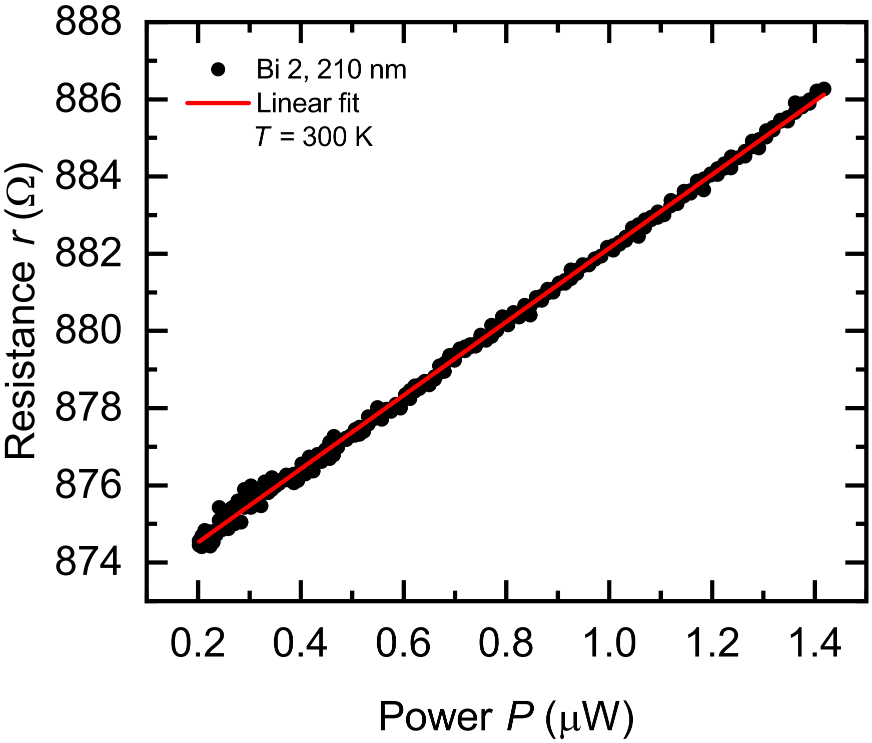

FIG. 2. **Resistance change of a Bi nanowire due to self-heating.** **a**, Four-terminal resistance r of Bi 2 (210 nm) as a function of the power P in the nanowire due to the applied current at $T = 300$ K. The slope of the red line (linear fit) yields the relation between r and P that is used to determine the thermal conductivity.

III. S3: PROCESS OF LATTICE RELAXATION

The proposed process of lattice relaxation is as follows. For Bi/TiO_2 3 (590, nm), the change of the electrical conductivity occurred at a bath temperature of 147 K. Two *I*-*U* curves of this nanowire were taken at this bath temperature which are given in Fig. 3. The resulting four-terminal resistance R of the second measurement is 19 times smaller than the resistance of the first measurement. Three minutes elapsed between both measurements. However, the measurement setup was not changed before, during or after the resistance change. As a result of the lattice relaxation, the semiconducting behavior of the electrical conductivity, which was induced by the strain effect of the shell, changed back to the semimetallic state, the original state of the Bi nanowires without a shell. This semiconducting to semimetallic transition also leads to a significant reduction of the absolute Seebeck coefficient.

The strain release can be attributed to the different thermal expansion coefficients of Bi ($\alpha_{\mathrm{Bi,c\text{-}axis}} \approx 17.4 \cdot 10^{-6}\,\mathrm{K}^{-1}$) at room temperature [7] and of TiO_2 ($\alpha_{\mathrm{TiO_2,c\text{-}axis}} = 8.4 \cdot 10^{-6}\,\mathrm{K}^{-1}$ up to $\alpha_{\mathrm{TiO_2,c\text{-}axis}} = 9.4 \cdot 10^{-6}\,\mathrm{K}^{-1}$) at room temperature [8]. As a result, the contraction of the Bi core is estimated to $\Delta d \approx 2.5\,\mathrm{nm}$ at 147 K compared to the original diameter during the manufacturing process. This leads to the irreversible lattice relaxation.

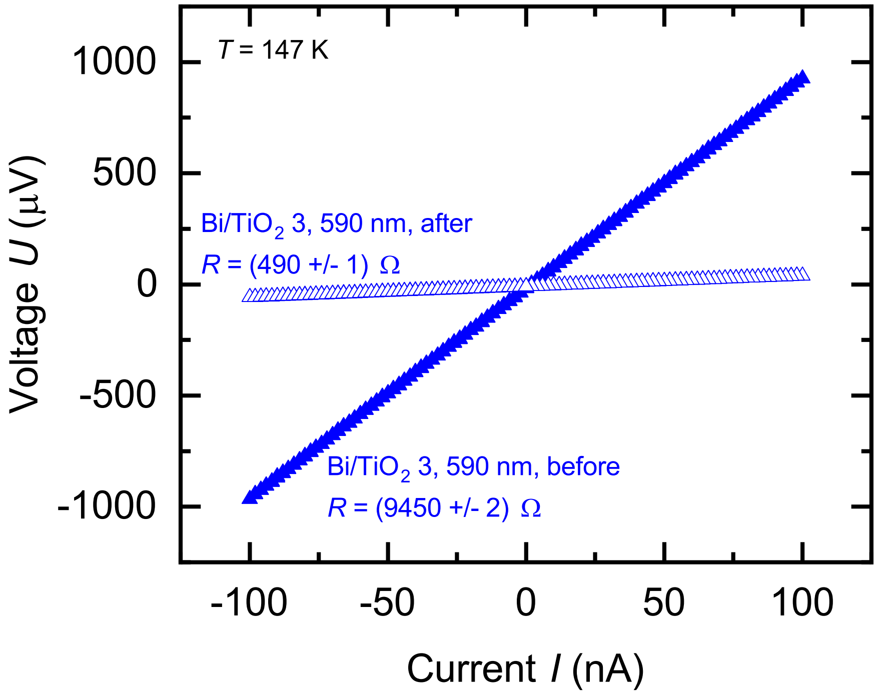

FIG. 3. **Resistance change of Bi/TiO_2 3 (590, nm) due to lattice relaxation.** Measured voltage U of the nanowire before and after the lattice relaxation as a function of the applied current I. The four-terminal resistance R of the nanowire is after the lattice relaxation 19 times smaller than before.

IV. S4: RESULTS AND DISCUSSION

A. Structural properties of Bi/Te nanowires

The distinct interface between the tellurium (Te) shell and the bismuth (Bi) core of Bi/Te nanowire (Bi/Te 1 (370 nm)) can been seen in the conventional transmission electron microscopy image in Fig. 4a. Elemental line scan obtained across the Bi/Te nanowire by energy dispersive X-ray (EDX) spectroscopy is given in Fig. 4b. The tellurium shell distribution of the Bi/Te core/shell nanowire (Bi/Te 1 (370 nm)) is not uniform which indicates the different Te shell thickness on both sides of the nanowire (also see the image obtained by scanning transmission electron microscopy below the EDX line scan). A non-uniform shell can be achieved when the nanowire is not completely perpendicular to the growth substrate during the sputtering process of the shell. In this case the nanowire shadowing effect with respect to the Te adatoms results in formation of a shell with a non-uniform thickness. The influence of the non-uniform shell on the thermoelectric transport properties is discussed later.

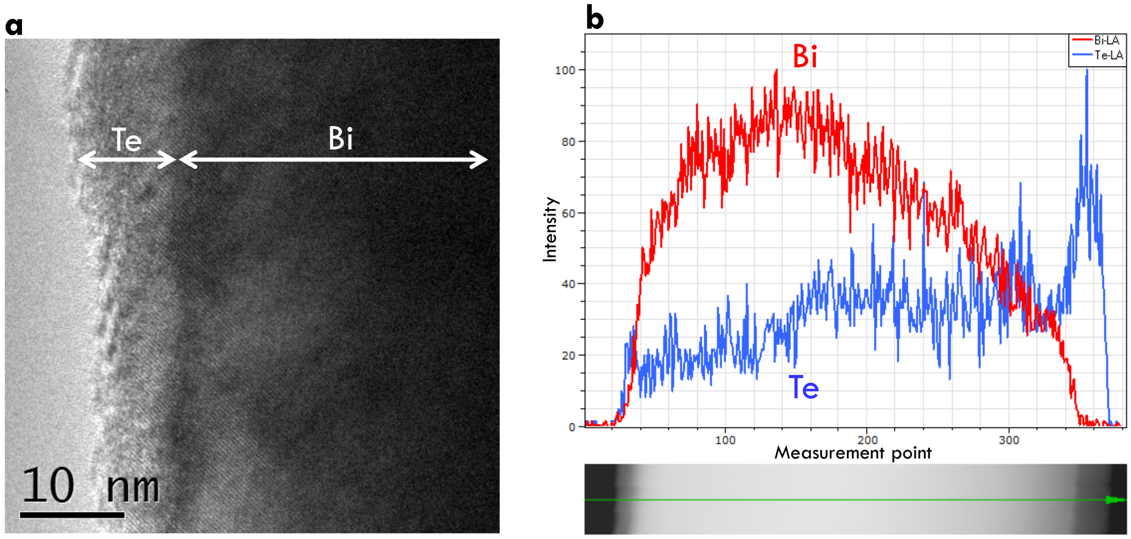

FIG. 4. **Structural properties of the Bi/Te core/shell nanowires.** **a**, Conventional transmission electron microscopy image that shows a section of the Bi/Te nanowire (Bi/Te 1 (370 nm)). **b**, Energy dispersive X-ray spectroscopy presenting the tellurium shell distribution of the Bi/Te core/shell nanowire (Bi/Te 1 (370 nm)).

Sample	Diameter d (nm)	Length l (μm)	Shell thickness t (nm)
Bi/Te 1	370 ± 5	21.7 ± 0.4	$10 - 30$
Bi/Te 2	490 ± 20	12.9 ± 0.8	$10 - 30$
Bi/Te 3	490 ± 10	12.9 ± 0.6	$10 - 30$

TABLE I. **Geometry parameters.** Overview of entire diameter d, length l and shell thickness t of bismuth/tellurium (Bi/Te) nanowires. Bi/Te nanowires are coated with a non-uniform Te-shell with a thickness of 10 nm − 30 nm by radio frequency sputtering. The geometry parameters have been determined by scanning and transmission electron microscopy.

B. Electrical properties

Fig. 5a shows the electrical conductivity σ of the Bi/Te nanowires as a function of the bath temperature T. Moreover, σ_{bulk} (perpendicular to the trigonal axis) from Ref. [1] is added to the diagram. Like for the Bi nanowires, the Bi/Te core/shell nanowires show also a reduced electrical conductivity compared to the bulk material. Moreover, the shell material can have an additional influence on the electrical conductivity. Kim *et al.* showed that the electrical conductivity of Bi/Te nanowires can be further reduced compared to Bi nanowires and to the bulk material due the compressive strain effect of Te shell on the Bi core [3, 4]. This was observed for Bi/Te nanowires with a uniform Te

shell thickness. However, the Bi/Te nanowires with a non-uniform shell thickness, which were investigated in this work, showed a reduction of the electrical conductivity compared to the bulk material that is in general not as large as for the Bi/Te nanowires with a uniform Te shell. This can be attributed to the strain effect of the shell on the core that will be larger for a uniform shell thickness than for a non-uniform shell. Furthermore, bismuth and tellurium are both conductive materials. As a result, the electrical conductivity has to be considered as parallel conduction in both materials. The total electrical conductivity of such a combination can be written as

$$\sigma_{\text{tot}} = \frac{A_{\text{Bi}}\sigma_{\text{Bi}} + A_{\text{Te}}\sigma_{\text{Te}}}{A_{\text{Bi}} + A_{\text{Te}}}. \tag{2}$$

A_{Bi} and A_{Te} are the cross-sectional areas of Bi and Te, respectively. σ_{Bi} and σ_{Te} are the partial conductivities of Bi and Te, respectively. The influence of the Te shell thickness on the reduction of the total electrical conductivity σ_{tot} is illustrated in the following example. Taking Eq. 2 and the electrical conductivity of Bi bulk $\sigma_{\text{Bi,bulk}} = 901600\,\Omega^{-1}\text{m}^{-1}$ [1] and Te bulk $\sigma_{\text{Te,bulk}} = 185\,\Omega^{-1}\text{m}^{-1}$ [5] and assuming the cross-sectional area of a nanowire with a total diameter of 300 nm results in a reduction of σ_{tot} by 13% compared to $\sigma_{\text{Bi,bulk}}$ if the Te shell thickness is 10 nm or in a reduction of σ_{tot} by 36% if the Te shell thickness is 30 nm. As a result, a uniform Te shell will lead to a larger reduction of σ compared to the bulk than a non-uniform shell.

C. Thermoelectric properties

Fig. 5b shows the absolute Seebeck coefficient S of the Bi/Te nanowires as a function of the bath temperature T. S_{bulk} (perpendicular to the trigonal axis) from Ref. [1] is added to the diagram. In general, for Bi/Te core/shell nanowires the direct influence of the Te shell on the total Seebeck coefficient as part of a parallel conduction model can be neglected due to the larger cross-sectional area and electrical conductivity of the Bi core compared to the Te shell. S of the Bi/Te core/shell nanowires is larger than that of Bi bulk. The absolute Seebeck coefficient of Bi/Te 1 (370 nm) is increased by 27 % compared to the bulk material at $T = 290\,\text{K}$ and it has the largest S of all investigated Bi-based nanowires in this work. This can be attributed to the compressive strain effect of the Te shell on the Bi core as previously reported in Ref. [3, 4]. However, S is smaller compared to the data given in Ref. [3, 4]. This can be explained by the non-uniform shell of the Bi/Te nanowires, as shown in Fig. 4a,b, and the resulting lower compressive strain effect of the Te shell on the Bi core compared to Bi/Te nanowires with a uniform shell.

D. Thermal properties

For the Bi/Te nanowires it was shown, that the rough interface between the Bi core and Te shell can lead to a reduction of the thermal conductivity as reported in Ref. [2–4]. However, the thermal conductivity of Bi/Te 1 (370 nm) is larger compared to other Bi/Te core/shell nanowires reported in Ref. [2–4]. This can be attributed to the non-uniform Te shell as shown in Fig. 4a,b. As a result, the compressive strain effect is lower compared to Bi/Te nanowires with a uniform shell. This will lead to a larger charge carrier contribution to the thermal conductivity increasing the overall thermal conductivity. Fig. 5c shows the thermal conductivity λ of the Bi/Te nanowires as a function of the bath temperature T. Moreover, λ_{bulk} (perpendicular to the trigonal axis) from Ref. [1] is added to the diagram.

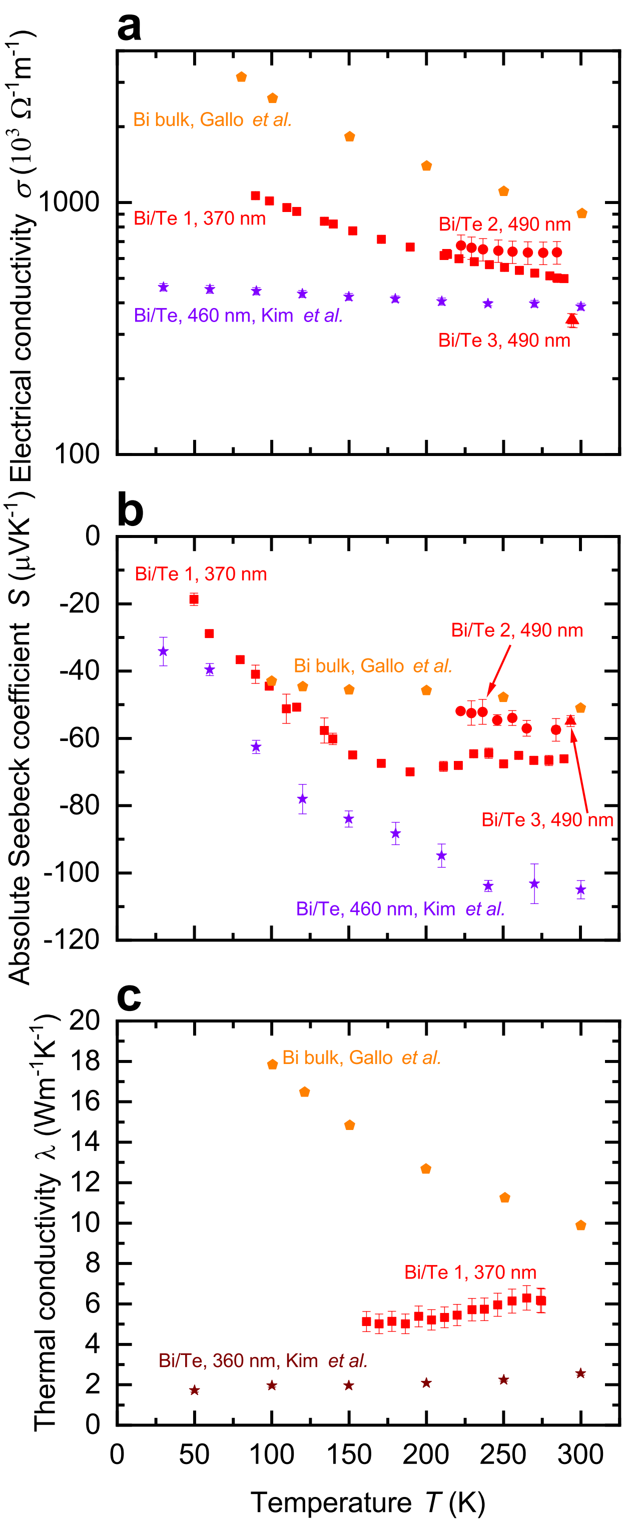

FIG. 5. **Electrical conductivity, absolute Seebeck coefficient and thermal conductivity of the Bi/Te core/shell nanowires with non-uniform Te shell.** **a**, Electrical conductivity σ of the Bi/Te core/shell nanowires as a function of the bath temperature T. σ is reduced compared to the bulk material but increased compared to a Bi/Te nanowire with a uniform Te shell. **b**, Absolute Seebeck coefficient S of the Bi/Te core/shell nanowires as a function of the bath temperature T. The modulus of S is increased compared to the bulk material but decreased compared to a Bi/Te nanowire with a uniform Te shell. **c**, Thermal conductivity λ of the Bi/Te core/shell nanowires as a function of the bath temperature T. The thermal conductivity of the Bi/Te nanowires is reduced compared to the bulk material and exhibits an opposite temperature dependence. The transport properties of Bi bulk (perpendicular to the trigonal axis) from Ref. [1] and of different Bi/Te nanowires with a uniform shell from Ref. [4] are added to the corresponding diagram. The Seebeck coefficient of the of Bi/Te nanowire from Ref. [4] was corrected by the absolute Seebeck coefficient of the reference material.

V. REFERENCES

[1] C. F. Gallo, B. S. Chandrasekhar and P. H. Sutter, *Journal of Applied Physics*, 1963, **34**, 144.
[2] J. Kang, J. W. Roh, W. Shim, J. Ham, J.-S. Noh and W. Lee, *Advanced Materials*, 2011, **23**, 3414.
[3] J. Kim, G. Kim, J.-H. Bahk, J.-S. Noh and W. Lee, *Nano Energy*, 2017, **32**, 520.
[4] J. Kim, M.-W. Oh, G. Kim, J.-H. Bahk, J. Y. Song, S. G. Jeon, D. W. Chun, J.-H. Bae, W. Shim and W. Lee, *Acta Materialia*, 2018, **144**, 145.
[5] A. Nussbaum, *Physical Review*, 1954, **94**, 337.
[6] F. Völklein, H. Reith. T. W. Cornelius, M. Rauber and R. Neumann, *Nanotechnology*, 2009, **20**, 325706.
[7] E. F. Cave and L. V. Holroyd, *Journal of Applied Physics*, 1960, **31**, 1357.
[8] D. R. Hummer, P. J. Heaney and J. E. Post, *Powder Diffraction*, 2007, **22**, 352.
